# Striatal hyperechogenicity as an ultrasound imaging marker for prodromal X-linked dystonia-parkinsonism

**DOI:** 10.1038/s41531-026-01418-4

**Published:** 2026-06-03

**Authors:** Martje G. Pauly, Cid Czarina E. Diesta, Paulo Cataniag, Max Borsche, Henrike Hanssen, Jean Quint L. Oropilla, Uwe Walter, Dirk Dressler, Shela Marie Algodon, Ana Westenberger, Christine Klein, Norbert Brüggemann

**Affiliations:** 1 Section for Movement Disorders, Department of Neurology, University of Luebeck and University Hospital Schleswig-Holstein Campus Luebeck, Luebeck, Germany; 2 Institute of Neurogenetics, University of Luebeck, Luebeck, Germany; 3 MakatiMed Institute of Neurological, Neurosurgical and Behavioral Sciences (M.I.N.D.S.), Makati, Philippines; 4 Department of Neurology, Rostock University Medical Center, Rostock, Germany; 5 German Center for Neurodegenerative Diseases (DZNE) Rostock/Greifswald, Rostock, Germany; 6 Movement Disorders Section, Department of Neurology, Hannover Medical School, Hannover, Germany

**Keywords:** Diseases, Genetics, Neurology, Neuroscience

## Abstract

X-linked dystonia-parkinsonism (XDP) is a neurodegenerative genetic disorder with striatal pathology. We investigated 138 participants (61 patients with XDP, 19 non-manifesting carriers (NMC), and 58 healthy controls (HC)) with transcranial sonography (TCS) to determine the hyperechogenicity of the lentiform nucleus (LN+), the size of the substantia nigra, and the width of the lateral and third ventricles. LN+ was correlated with LN volume as measured by structural T1 imaging. Hexameric repeat number within the causative insertion was determined as a potential modifier. The prevalence of LN+ was higher in patients with XDP (81%) and in NMC (47%) compared to HC (20%). In NMC and XDP with LN+, the estimated age at onset was younger, and the repeat number was higher. There was no difference in the size of the substantia nigra nor in the width of the lateral ventricle. The width of the third ventricle was higher in patients with XDP and correlated with age at examination and disease duration. The MRI-derived LN volume was higher in HC than in NMC and XDP. There were no volume differences between LN+ and LN−. LN+ is observed more frequently in patients with XDP and even several years before symptom onset in NMC, particularly in those with a high genetic modifier burden. TCS might therefore be a helpful tool to identify persons at risk for a more imminent disease manifestation among the NMCs.

## Introduction

X-linked dystonia-parkinsonism (XDP) is a neurodegenerative disorder mainly affecting males of Filipino descent^1,2^. First signs are typically focal dystonia with rapid generalization and parkinsonism occurring in later disease stages^2^. The cause of the disorder is an insertion of a short interspersed element [SINE]–variable number of tandem repeats [VNTR]–Alus (SVA) retrotransposon in intron 32 of the *TAF1* gene^3–5^. While most patients start developing symptoms around the age of 40 years^2^, there is a broad range of ages of onset (AAO). AAO is significantly determined by four currently recognized XDP genetic modifiers, i.e., repeat number of a (CCCTCT)_n_ repeat^3,5^ within the SVA retrotransposon insertion and modifiers tagged by three single-nucleotide polymorphisms (SNPs)^6^. Even before the manifestation of motor symptoms, non-manifesting carriers (NMC) of the SVA retrotransposon insertion may exhibit subtle deficits in oculomotor function^7^, subclinical changes of movements^8^, and slight atrophy of the striatum and pallidum^9^, indicating neurodegeneration prior to the manifestation of clinically overt signs. Different studies utilizing post-mortem examinations^10,11^, magnetic resonance imaging (MRI)^12,13^, single-photon emission computed tomography^14^, electroencephalography^15–17^, transcranial magnetic stimulation^18^, and tracking of eye movements^19^, identified the striatum as the key localization of pathology.

Transcranial sonography (TCS) is a non-invasive technique to investigate basal ganglia and midbrain structures in movement disorders^20^. In Parkinson’s disease (PD), an increased size of echogenic area (‘hyperechogenicity’) of the substantia nigra (SN) can be detected in approximately 90% of patients^21^. The hyperechogenicity already occurs in the prodromal phase^22^, such as in persons with isolated hyposmia or idiopathic REM sleep disorder^23^, and does not seem to change in size over the course of the disease^21,24–28^. A hyperechogenicity of the SN (SN+), lentiform nucleus (LN+), and caudate nucleus can also be found in Huntington’s disease^29,30^, correlating with motor symptoms^31^. Also, in patients with atypical parkinsonian disorders^24^ and Wilson’s disease^32^, additional hyperechogenicity can be detected in the striatum, highlighting that the pathological changes in these diseases extend beyond the SN. In dystonias, LN+ has also been described^33–35^. However, LN+ differs based on the localization of dystonia^33,35,36^ and the genetic cause^34,37,38^.

A previous TCS study investigating patients with XDP, first-degree relatives, and controls found that the majority of patients exhibited SN+ and LN+, with higher frequencies of SN+ in patients with a predominant parkinsonian phenotype^39^.

Here, we investigated 138 male Filipino participants using TCS, clinical scores, and genetic testing to further elucidate the relationship between TCS abnormalities, clinical features, and genotype. This study is intended to validate the ultrasound findings of the earlier investigation in a significantly larger number of patients (61 vs. 39), NMC (19 vs. 7) and matched Filipino healthy controls (HC, 58 vs. 30). A particular aspect that was not possible in the previous study is whether genetic modifiers, have an influence on TCS features and whether TCS could be used as a marker for NMC with a more imminent risk to convert to overt XDP. Since striatal neurodegeneration is already pronounced at XDP disease onset, NMC exhibiting striatal pathology are likely optimal candidates for disease-modifying treatments.

## Results

### Demographic and clinical observations

**Fig. 1:**
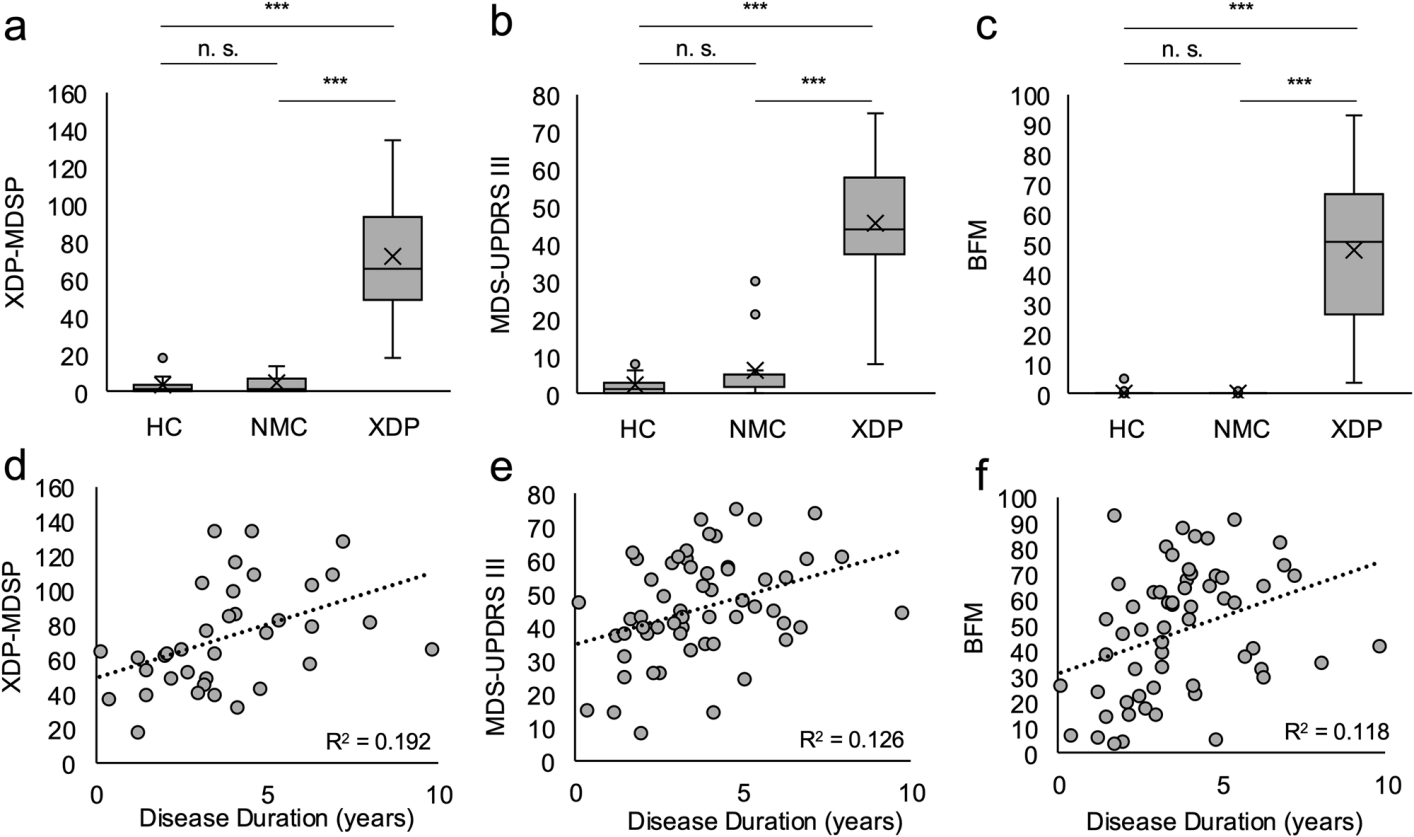
Clinical scores. Clinical scores with respect to the group showing the 25th to 75th percentile as a box, the median as a line, the mean as an X, the minimum and maximum value in the range of 1.5× interquartile range as whiskers, and values outside of the 1.5× interquartile range as dots. **a** The X-linked dystonia-parkinsonism (XDP) Movement Disorder Society of the Philippines Rating Scale (XDP-MDSP RS) of healthy controls (HC), non-manifesting carriers (NMC), and patients with XDP. **b** The Movement Disorder Society-sponsored revision of the Unified Parkinson’s Disease Rating Scale Part III (MDS-UPDRS III) for HC, NMC, and patients with XDP. **c** The motor part of the Burke–Fahn–Marsden Dystonia Rating Scale (BFMDRS) for HC, NMC, and XDP. Correlation between disease duration **d** XDP-MDSP RS, **e** MDS-UPDRS III, and **f** BFMDRS in XDP patients. ***p-value < 0.001; n.s. not significant. Line: trend line.

We included 61 patients with XDP, 19 NMC, and 58 HC in our study. The AAE was higher in the XDP group than in the NMC and HC groups, but similar comparing NMC and HC (for details see Table 1). This difference was taken into consideration for further analyses. The estimated AAO (eAAO) was similar in the XDP and NMC group (Table 1). There was no difference in the clinical scores (Movement Disorder Society-sponsored revision of the Unified Parkinson’s Disease Rating Scale (MDS-UPDRS)^40^, Burke–Fahn–Mardsen Score (BFMDS)^41^, XDP score of the Movement Disorder Society of the Philippines (XDP-MDSP RS)^42^) between HC and NMC, which means that NMC exhibited no observable motor phenotype (Fig. 1A–C, Table 1). Patients with XDP usually showed both dystonic and Parkinsonian signs of the disease, with all three clinical assessments correlating with AAE (XDP-MDSP RS: Pearson Correlation = 0.328, *p*-value = 0.006; MDS-UPDRS: Pearson Correlation = 0.389, *p*-value < 0.001; BFMDRS: Pearson Correlation = 0.241, *p*-value = 0.048). All three clinical assessments also correlated with disease duration (DD) when corrected for age at examination (AAE) (XDP-MDSP RS: Correlation = 0.524, *p*-value = 0.02; MDS-UPDRS: Correlation = 0.512, *p*-value = 0.025; BFMDRS: Correlation = 0.600, *p*-value = 0.003) (Fig. 1D–F).

**Table 1:** Demographic, clinical, and ultrasound information

	XDP *n* = 61	NMC *N* = 19	HC *n* = 58	*p*-values*
**Demographic information**				
AAE (y)	40.3 (7.0)	34.4 (5.9)	33.7 (7.5)	<0.001/0.569/0.009
AAO (y)	36.6 (7.1)	-	-	-/-/-
eAAO (y)	37.6 (7.0)	39.7 (8.2)	-	-/-/0.282
DD (y)	3.7 (2.9)	-	-	-/-/-
eDD (y)	2.7 (5.2)	−5.3 (7.7)	-	-/-/<0.001
**Clinical information**				
XDP-MDSP RS	72.8 (30.4)	4.1 (5.8)	2.7 (3.5)	<0.001/0.939/<0.001
MDS-UPDRS III	45.7 (15.6)	6.1 (9.1)	1.7 (2.0)	<0.001/0.324/<0.001
BFMDRS	47.8 (24.8)	0.3 (0.8)	0.4 (1.0)	<0.001/0.958/<0.001
**Ultrasound information**				
LN+	48/59 (81.1%)	8/17 (47.1%)	11/54 (20.4%)	<0.001/0.030/0.010
SN+_max_ (cm^2^)	0.14 (0.06)	0.13 (0.05)	0.11 (0.04)	0.195
Path. SN+max	12/59 (20.3%)	2/17 (11.8%)	1/56 (1.8%)	0.002/0.069/0.422
Third ventricle (mm)	4.94 (1.2)	3.69 (2.7)	2.69 (1.3)	<0.001/0.063/<0.001
Lateral ventricle (mm)	17.1 (1.9)	16.7 (1.7)	16.7 (2.0)	0.399

Parenthesis: standard deviation; *p-values given for HC and XDP/HC and NMC/NMC and XDP.

*XDP* X-linked dystonia-parkinsonism, *NMC* non-manifesting carrier, *HC* healthy controls, *AAE* age at examination, *y* years, *AAO* age at onset, *eAAO* estimated age at onset, *DD* disease duration, *eDD* estimated disease duration, *XDP-MDSP RS* XDP Movement Disorder Society of the Philippines Rating Scale, *MDS-UPDRS III* movement disorder society-sponsored revision of the Unified Parkinson’s Disease Rating Scale Part III, *BFMDRS motor* motor part of the Burke–Fahn–Mardsen Dystonia Rating Scale. *LN+* Hyperechogenicity of the lentiform nucleus, number after/indicates *n* for the specific variable, *SN+max* maximum size of hyperechogenicity of the substantia nigra, *Path* pathological SN+max with a size >0.2 cm^2^.

### Transcranial sonography

#### Temporal bone window and data availability

Data on LN+ were available for 59 patients with XDP (96.7%), 17 NMC (89.5%), and 54 HC (93.1%). Data on SN+max were available for 59 patients with XDP (96.7%), 17 NMC (89.5%), and 56 HC (96.6%). Data on the width of the third ventricle were available for 61 patients with XDP (100%), 18 NMC (94.7%), and 58 HC (98.3%). Data on the width of the lateral ventricles were available for 39 patients with XDP (63.9%), 17 NMC (89.5%), and 39 HC (67.2%). TCS was not possible due to a poor bone window in one patient with XDP (1.6%), one NMC (5.2%), and two HC (3.4%).

#### Hyperechogenicity of the lentiform nucleus

**Fig. 2:**
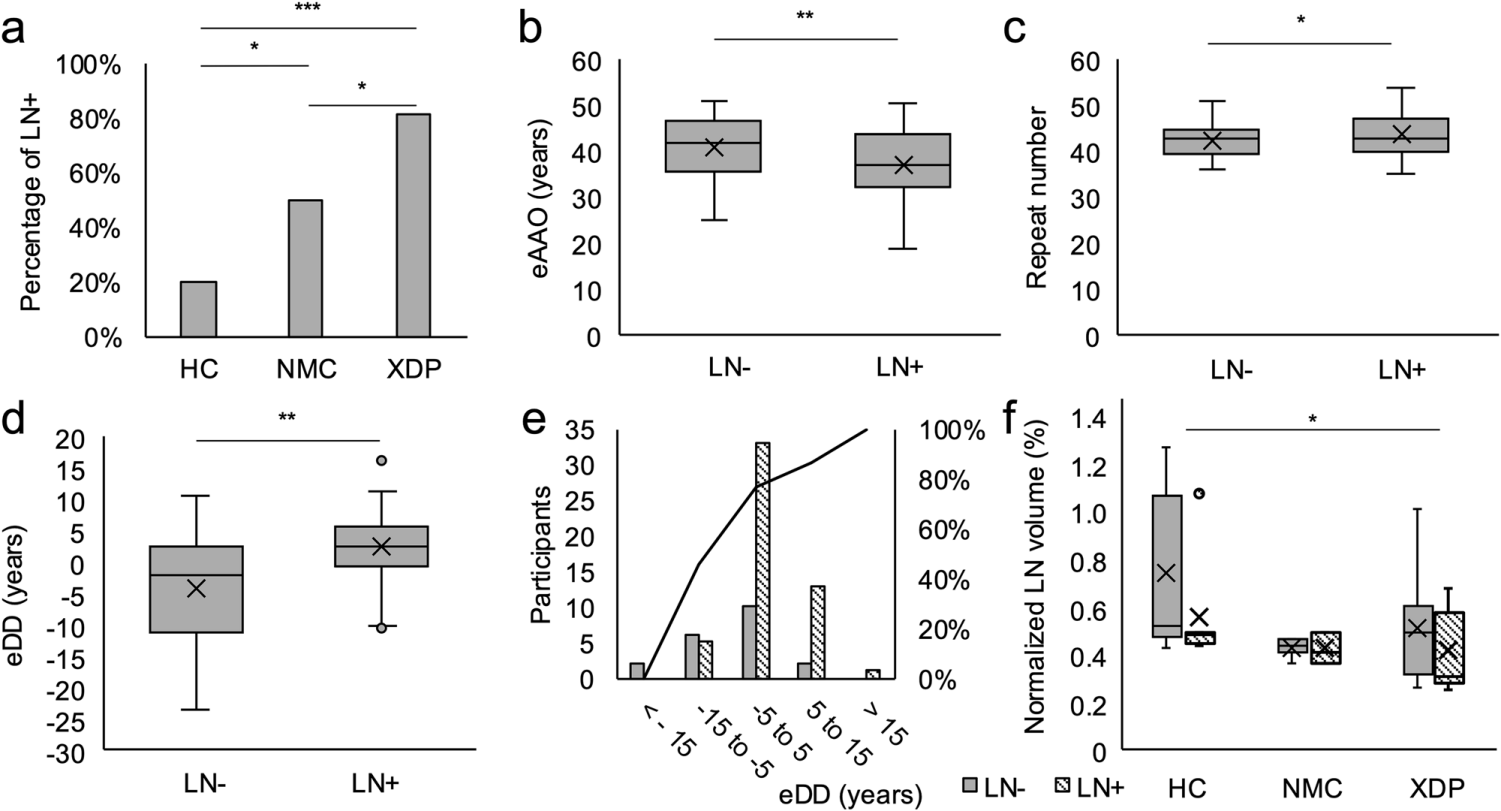
Hyperechogenicity of the lentiform nucleus. **a** Percentage of participants with hyperechogenicity of the lentiform nucleus (LN+) in the group of healthy controls (HC), non-manifesting carrier (NMC), and patients with X-linked dystonia-parkinsonism (XDP), **b** estimated age at onset (eAAO) in the group with LN+ and without hyperechogenicity (LN-), **c** repeat number in LN+ and LN−, **d** estimated disease duration (eDD) in LN+ and LN−, **e** Number of participants with LN+ and LN- according to grouped eDD (left y axis) and percentage of participants with LN+ according to grouped eDD (right y axis), **f** normalized LN volume according to group and LN+ status. **a**–**d** and **f** variable are shown with the 25th to 75th percentile as a box, the median as a line, the mean as an X, the minimum and maximum value in the range of 1.5× interquartile range as whiskers, and values outside of the 1.5× interquartile range as dots. ***p-value < 0.001; **p-value < 0.01; *p-value < 0.05.

In the XDP group, 48 out of 59 participants (81.1%) showed LN + . The numbers were lower in the NMC group with 8 out of 17 individuals (47.1%) and in the HC group, with 11 out of 54 individuals (20.4%) demonstrating LN+ (Fig. 2A). The group had a significant effect on the occurrence of LN+ (*χ*²(3) = 45.58, *p*-value = <0.001). The model explained 39% of the variance (Nagelkerke *R*² = 0.394) and showed good fit (Hosmer–Lemeshow *χ*²(8) = 6.44, *p*-value = 0.598). XDP had 15.30 times higher odds of LN+ (OR = 15.30, 95% CI [5.69–41.12], *p*-value: <0.001), and NMC had 3.45 times higher odds of LN+ (OR = 3.45, 95% CI [1.08–11.01], *p*-value = 0.037) compared to HC. In a direct comparison between NMC and XDP, NMC had lower odds of the LN+ (OR = 0.23, 95% CI [0.07–0.75], *p*-value = 0.015), corresponding to XDP having approximately 4.4 times higher odds than NMC. AAE was not a significant predictor (OR = 1.02, 95% CI [0.96–1.08], *p*-value = 0.557).

Comparing NMC and patients with XDP with LN+ and without hyperechogenicity (LN−) corrected for AAE, the eAAO was lower in the group with LN+ (LN−: 40.8 ± 6.8, LN+: 37.3 ± 7.1, *p*-value = 0.003) (Fig. 2B), the repeat number was higher (LN−: 42.4 ± 3.8, LN + : 43.8 ± 4.8, *p*-value = 0.040) (Fig. 2C), and the estimated DD (eDD) was longer (LN−: −4.0 ± 8.7, LN+: 2.05 ± 5.3, *p*-value = 0.003) (Fig. 2D+E). There was no difference between LN− and LN+ for AAO, DD, and clinical scores.

The volume given as a percentage of the total intracranial volume of LN based on MRI differed between groups (*p*-value = 0.012), but not between LN+ and LN− (*p*-value = 0.182). LN volume was higher in HC (0.67% ± 0.29) than in NMC (0.41% ± 0.04, *p*-value = 0.006) and in XDP (0.43% ± 0.18, *p*-value < 0.001). There was no difference in LN volume between XDP and NMC (*p*-value = 1.00) (Fig. 2F).

#### Hyperechogenicity of the substantia nigra

SN+_max_ did not differ between groups (*p*-value = 0.195) and was 0.11 ± 0.04 cm^2^ (range: 0.00–0.26 cm^2^) in HC, 0.13 ± 0.05 cm^2^ (0.08–0.25 cm^2^) in NMC, and 0.14 ± 0.06 cm^2^ (0.05–0.29 cm^2^) in patients with XDP (Supplementary Fig. 1A). There was also no difference (*p*-value = 0.087) between HC and variant carriers (NMC and XDP combined) with SN+_max_ being 0.13 ± 0.05 cm^2^ (0.05–0.29 cm^2^). The percentage of uncertain pathogenic (0.2–0.24 cm^2^) and pathogenic (>0.24 cm^2^) SN+_max_, however, differed between groups (*p*-value = 0.037) with seven probands with uncertain pathogenetic SN+_max_ and five pathogenic SN+_max_ in XDP, one and one in NMC, and none and one in HC, respectively (Supplementary Fig. 1B). The rate of SN+_max_ ≥0.2 was only different between XDP and HC (*p*-value = 0.02) but not between XDP and NMC (*p*-value = 0.422). However, there was a trend for higher frequency in NMC compared to HC (*p*-value = 0.069) (Supplementary Fig. 1C). There was no difference between patients with XDP who had normal and pathogenic SNmax+ regarding DD and clinical scores.

#### Measurements of the ventricular system

**Fig. 3:**
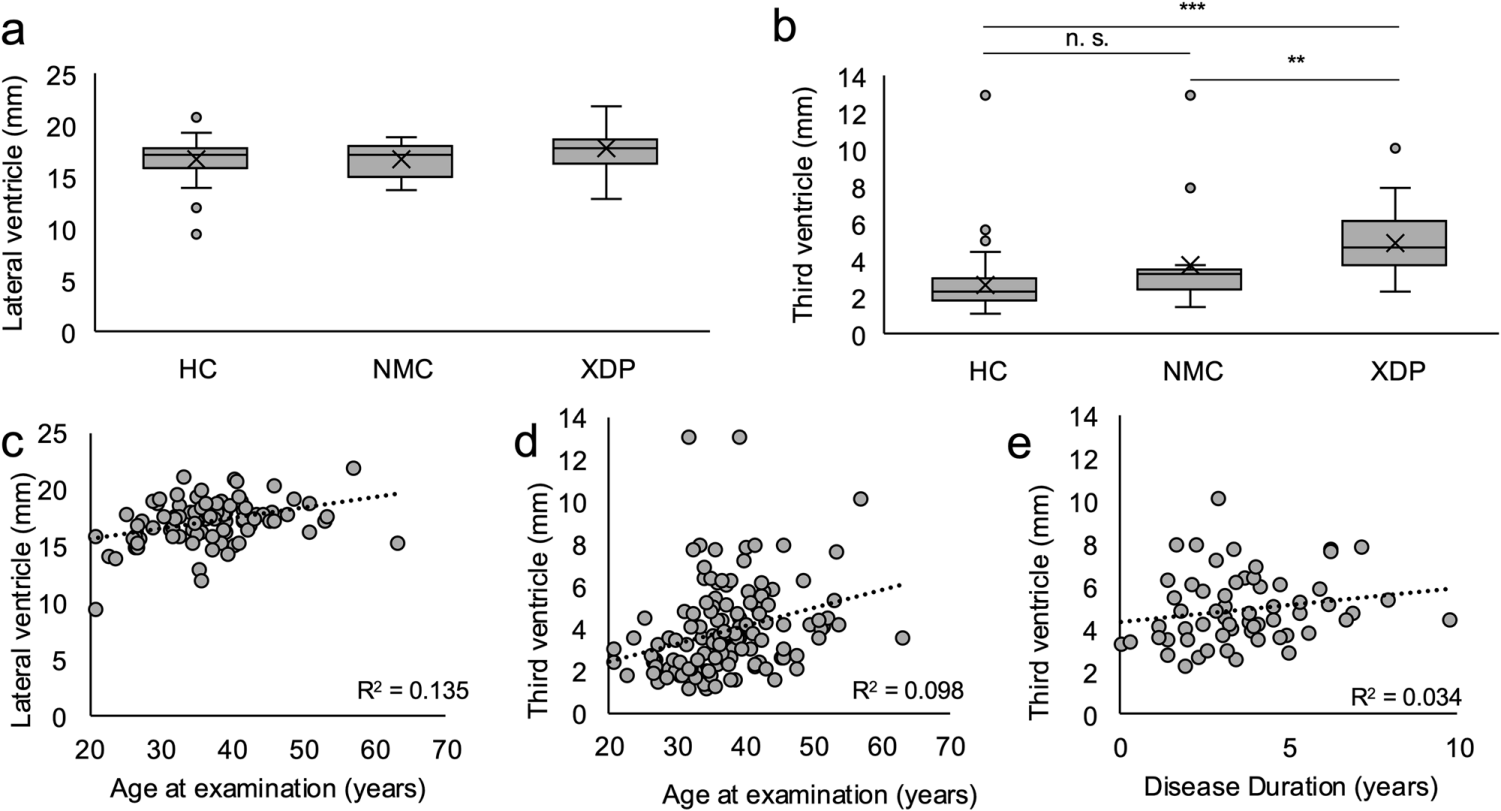
Third and lateral ventricles. Width of lateral ventricle and third ventricle in regard to the group showing the 25th to 75th percentile as a box, the median as a line, the mean as an X, the minimum and maximum value in the range of 1.5× interquartile range as whiskers, and values outside of the 1.5× interquartile range as dots. **a** The width of the lateral ventricles in mm of healthy controls (HC), non-manifesting carriers (NMC), and patients with X-linked dystonia-parkinsonism (XDP). **b** The width of the third ventricle of HC, NMC, and patients with XDP. Correlation between **c** the width of the lateral ventricles and age at examination, **d** the width of the third ventricle and age at examination, and **e** the width of the third ventricle and disease duration. ***p-value < 0.001; **p-value < 0.01; n.s. not significant. Line: trend line.

There was no difference in lateral ventricles between groups (*p*-value = 0.399) with the width of lateral ventricles being 17.1 ± 1.9 mm in patients with XDP, 16.7 ± 1.7 mm in NMC, and 16.7 ± 2.0 mm in HC (Fig. 3A). The width of the third ventricle was larger in patients with XDP (4.94 ± 1.16 mm) compared to HC (2.69 ± 1.27 mm, *p*-value < 0.001) and NMC (3.69 ± 2.70 mm, *p*-value = 0.001). The *p*-value for the difference between HC and NMC did not reach the cutoff for significance (*p*-value = 0.063) (Fig. 3B). The AAE correlated with both the width of lateral ventricles (*r*: 0.367, *p*-value = 0.001) and the width of the third ventricle (*r*: 0.313, *p*-value = 0.001) (Fig. 3C, D). The width of the third ventricle correlated with the DD, corrected for AAE (rho: 0.486, *p*-value = 0.04) (Fig. 3E).

#### Longitudinal analysis

Longitudinal data were available for 11 participants with measurements in 2019 and 2024, respectively. In 2019, four participants were classified as patients with XDP, two as NMC, and five as HC. None of the NMC developed symptoms prior to the examination in 2024. Mean changes in XDP-MDSP RS were 2.8 ± 3.6, 4.0 ± 2.8, and 12.0 ± 24.1, respectively. Mean increase in MDS-UPDRS III was 1.4 ± 2.3, 7.0 (only available for one NMC), and 11.0 ± 4.2. Mean increase in BFM was 0.0 ± 0.0, 1.0 ± 1.4, and 11.9 ± 45.2, respectively. One out of two NMCs with data available showed LN+, and all patients with XDP showed LN+ (data for all four available). The NMC with LN+ had a larger increase in XDP-MDSP RS (from 0 to 6) than the NMC with LN− (from 0 to 2). One of the HC (data available for four HC) was classified as LN+ in 2019, but not in 2024 due to technical reasons, and only a moderate bone window. There was no change in LN+ between 2019 and 2024 in the other participants. Mean increase in width of the third ventricle was 0.02 ± 0.77 mm in HC, 0.70 ± 0.28 mm in NMC, and 1.03 ± 0.61 mm in patients with XDP.

## Discussion

In this study, we showed that, while there was no difference in clinical scores between NMC and HC, the prevalence of hyperechogenicity of the lentiform nucleus was higher in NMC compared to HC, as well as in patients with XDP compared to NMC and HC. Patients with XDP and NMC showing LN+ appeared to have a more severe ‘genetic burden’ based on hexameric repeat length and genotypes related to XDP AAO modifiers. Furthermore, we found an increased subcortical atrophy in patients with XDP compared to controls, as demonstrated by a more pronounced and progressively increased width of the third ventricle.

LN+ is not only found in patients with movement disorders, but can also be identified in healthy people. The percentage of LN+ in the healthy control groups ranges from 1.4%^43^ to 20%^44^, with a single study reporting up to 50%^45^. However, most studies found LN+ in under 15% of healthy controls^24,36,46^. Additionally, none of the previous studies have been performed in participants of Filipino descent, so this is the first time that a percentage of 20% of LN+ has been reported in this population. Therefore, investigating LN+ in XDP, NMC, and HC to evaluate LN+ as a biomarker for XDP is a reasonable approach.

Age-related effects must nevertheless be taken into account. With respect to substantia nigra hyperechogenicity, our previous work demonstrated a slight age-related increase in area that, however, remains within the physiological range^47^. In contrast, the presence of LN+ is a qualitative marker that has mainly been reported in atypical Parkinsonian syndromes or Huntington’s disease (HD) and is usually absent even in classical Parkinson’s disease, which typically manifests later in life than XDP. A systematic review reported a prevalence of 16% in PD, comparable to that observed in our much younger healthy controls^26^. Moreover, other studies with control groups that were substantially older than the XDP and NMC cohorts in our study did not demonstrate elevated rates of LN+^24,36^. To further address the difference in age at examination as a potential confounder, we additionally performed a binary logistic regression analysis, which showed that AAE was not a significant predictor of LN+ (*p* = 0.557).

LN+ can typically be detected in patients with atypical Parkinsonian disorders^24,48^. These studies revealed LN+ in 66–100% of patients with progressive supranuclear palsy, in 57–75% of patients with multiple system atrophy, and in 86% of patients with corticobasal degeneration^24,48^. The mechanism underlying the occurrence of LN+, both in general and in atypical Parkinsonian disorders in particular, has not been fully understood. Similar to SN+, it might be associated with the accumulation of iron, which has been demonstrated in the pallidum and putamen in multiple system atrophy, progressive supranuclear palsy^26^, and XDP^12^. Other heavy metals may also contribute to increased echogenicity, as seen in Wilson’s disease, which is another disorder associated with LN+. In this condition, a correlation has been identified between LN+ and copper concentration in the putamen^32^. While HD is most commonly associated with SN+, a study investigating a subgroup of patients with a hypokinetic rigid phenotype found LN+ in more than 50% of patients. However, not all patients exhibited LN+ despite clear evidence of striatal neurodegeneration in this disorder^29^.

In a previous TCS study involving 37 male patients with XDP, as well as relatives and controls, 81% of patients showed LN+, which is consistent with our results^39^. The disease duration correlated with digitized echo intensity measures of LN^39^. Since we did not quantify the intensity or size of LN+, we were unable to investigate a correlation between disease duration and LN+. However, the percentage of probands with LN+ increased with eDD, starting 10–15 years prior to eAAO (Fig. 2E). Interestingly, genetic factors appear to influence LN+, with patients with XDP and NMC exhibiting LN+ having a more severe ‘genetic burden’. Namely, in the group with LN+, the eAAO based on the four XDP genetic modifiers was lower, and the eDD calculated as the difference between current age and eAAO was higher. Since there was no difference in the actual AAO, DD, or clinical scores, our findings suggest that genetic modifiers may impact LN+.

LN+ can also be observed in patients with idiopathic dystonia^33,36^, with those having cervical and upper limb dystonia showing more frequent LN+ than patients with facial dystonia, including blepharospasm and oromandibular dystonia^33,36^. With respect to genetic forms of dystonia, a study including five patients with dopa-responsive dystonia (DYT-*GCH1*) found no LN+^37^, while another study investigating 11 patients identified LN+ in seven patients^49^. No LN+ was found in three patients with DYT-*TOR1A*^34^. Of note, idiopathic and genetic forms of dystonia are, in most cases, not neurodegenerative and therefore a different disease mechanism may underlie LN+ in these cases.

Another main finding of the previous TCS XDP study was the presence of pathogenic SN+, defined for the TCS system as a size >0.2 cm^2^, which was observed in 79% of XDP patients^39^. In our study, only 20% of XDP patients had pathogenic SN+ when considering 0.2 cm^2^ as a cutoff. When taking a more conservative cutoff of 0.24 cm^2^, the percentage decreases to 11%. While there was no statistical difference in the size of the SN between HC, NMC, and patients with XDP, the percentage of pathogenic SN+ was higher in the XDP group than in HC. Of note, in the previous study, all investigated members of each family had either all SN+ or none at all, regardless of the genetic status, suggesting the presence of contributing factors other than the *TAF1* variant^39^. A similar phenomenon was found in a study investigating relatives of PD patients, in which 45% of relatives had SN+^50^. Here, SN+ was associated with mild clinical findings^50^. SN+ is typically associated with PD^24,51^, while multiple studies investigating SN+ in primary dystonia^36,45^ and DYT-*GCH1*^37^ found no differences to controls.

Overall, our findings of increased LN+ and no relevant difference in SN+ size are in line with the current understanding of the pathophysiology of XDP. MRI studies confirmed marked neurodegeneration predominantly in the striatum, followed by the pallidum^10,52^. Another study indeed found pathological uptake in dopamine transporter scintigraphy in three out of four patients^14^, which, however, was driven by the striatal atrophy. Another important finding is the increased iron accumulation in the striatum^12–14^, which may at least partially be causal for the hyperechogenicity observed in TCS. While some studies in patients with PD have reported a relationship between SN+ with iron deposition and neuromelanin loss^28^, another study did not find this association^53^. The exact mechanisms leading to SN+ and LN+ in TCS still remain to be elucidated.

Due to the small number of patients investigated longitudinally, only limited conclusions can be drawn from the data. Interestingly, the increase in width of the third ventricle was higher in patients with XDP and NMC than in HC. This may indicate increased atrophy over the five-year period. However, further studies involving more patients and additional imaging techniques, such as MRI, are necessary.

The main limitation of our study is the cross-sectional character, with only a small subset of participants assessed longitudinally, and no conversion from NMC to XDP observed in this group. Further longitudinal studies that follow NMC and HC clinically and apply TCS, and ideally also MRI, at multiple time points are needed to draw more precise conclusions regarding LN+ as an early indicator of clinical conversion. An important limitation in the interpretation of the volumetric analyses arises from the acquisition of T1-weighted images on different scanners and at varying magnetic field strengths. While FreeSurfer has demonstrated robustness across such acquisition settings^54^, systematic bias may still occur and should be carefully considered when interpreting the results, especially regarding the longitudinal analysis.

In conclusion, LN+ is more frequently observed in patients with XDP and several years prior to symptom onset in a subset of non-manifesting carriers. The genetic modifier burden was higher in participants with LN+. Longitudinal studies are needed to clarify the relationship between symptom onset and LN+, as well as to explore differences between patients with and without LN+.

## Methods

### Participants

Male participants of Filipino descent were included as part of multimodal studies performed at the University of Lübeck, Germany, in 2013 and 2014, prior to deep-brain stimulation surgery or at Makati Medical Center, Manila, Philippines, in 2019 and 2024. Of all 138 participants, 61 were clinically affected by XDP and genetically confirmed to carry the *TAF1* SVA retrotransposon insertion. The other participants were first-degree male relatives of XDP patients or one of the additional eleven Filipino controls without a family relation, who were locally recruited in Lübeck. All were neurologically healthy. Of these 77 participants, 19 carried the *TAF1* mutation and were classified as NMC, while the remaining 58 did not carry the variant and were classified as HC. All examiners were blinded regarding the genetic status of NMC and HC during the examination. In the case of 11 participants (four XDP, two NMC, and five HC), data from two time points (2019 and 2024) were available. For those 11 participants, data from 2019 were included for the overall analysis. The study was approved by both local ethics committees of the University of Lübeck (approval number 21-263) and the Makati Medical Center (approval number MMCIRB 2017-135). Prior to participation, all participants were informed about the study in Tagalog, the official language of the Philippines, and written informed consent was obtained. The study was conducted in accordance with the Declaration of Helsinki.

### Clinical examination

Clinical examinations were performed by movement disorder specialists. Parkinsonian features were separately assessed using part III of MDS-UPDRS^40^. Dystonia symptoms were assessed using the BFMDS^41^. In patients assessed in 2019 and 2024, the XDP-MDSP RS^42^ was used, assessing dystonic symptoms (part I), Parkinsonian symptoms (part II), non-motor features (part III), and activities of daily living (part IV). Part V (global impression) was not used for analysis in this study. In addition, information about AAO in XDP patients was collected, and the DD was calculated as the number of years between AAO and AAE.

### Genetic testing

All participants were genetically tested for the presence of the SVA retrotransposon insertion in *TAF1*^55^. Furthermore, the repeat number of (CCCTCT)_n_ repeats, the genotypes at the SNPs tagging the three genetic modifiers (rs245013, rs33003, rs62456190), and the eAAO were determined as previously described^3,5,6,56^. The eDD was calculated as the number of years between eAAO and AAE.

### Transcranial sonography

**Fig. 4:**
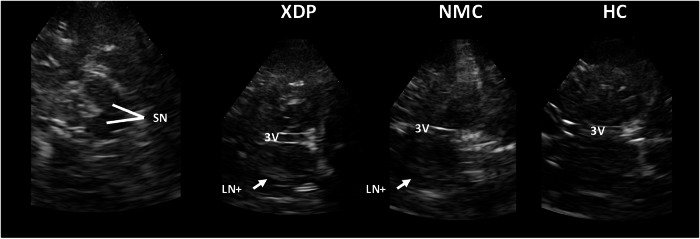
Transcranial sonography. Transcranial sonography imaging of the substantia nigra (SN), the third ventricle (3V), the lentiform nucleus with (LN+) or without hyperechogenicity (LN−) in patients with X-linked dystonia-parkinsonism (XDP), non-manifesting carriers (NMC), and healthy controls (HC).

TCS was performed in all participants by the same examiner (N.B.) using a Siemens Acuson Antares (2013/2014) or Esaote Mylab Alpha (2019/2024) device with corresponding 1–4 MHz sector probes. TCS was performed as previously described^39^: the midbrain was investigated via the temporal bone window in the axial plane. In TCS, the pallidum cannot be sufficiently distinguished from the putamen in spatial resolution, hence reference is made to the LN, as in previous studies. Qualitative assessment of the echogenicity of the LN was done on both sides. If either side demonstrated hyperechogenicity, defined as higher intensity than the surrounding white matter, the dichotomous variable LN+ was set to “yes”. If neither side showed hyperechogenicity, LN+ was set to “no”. Both right and left SN+ were measured in cm^2^, and the maximum value was used for further analysis as SN+_max_. SN+_max_ was considered uncertain pathogenic if it was between 0.2 and 0.24 cm^2^ and pathogenic if it was >0.24 cm^2^. The widths of the third ventricle and lateral ventricles were measured in mm. For lateral ventricles, both right and left cella media (pars centralis) of the lateral ventricles were measured, and the mean of both measurements was calculated (Fig. 4).

### Magnetic resonance imaging

Structural magnetic resonance imaging (MRI) data (high resolution T1-weighted images acquired using a 3-dimensional magnetization-prepared rapid acquisition gradient echo sequence) was available from previously published imaging studies from a total of 76 of the 138 participants (HC: 34, NMC: 10, XDP: 32). Forty-seven participants were examined in Manila using a 1.5 T Magnetom Aera, Syngo MR D13 (Siemens Healthcare, Erlangen, Germany) with a 20-channel head coil. In Lübeck, 29 participants were examined: 22 participants in a 3.0 T Achieva (Philips, Best, the Netherlands) with an 8-channel head coil and seven participants in a 3.0 T Ingenia (Philips) with a 32-channel head coil. The volume of the LN, considered as a combination of the pallidum and putamen, was calculated using the Freesurfer protocol^57,58^ and normalized for the total intracranial volume. FreeSurfer was chosen for its robustness and comparability across different scanners and field strengths^54^. For detailed information, see previous publications^12,13,54^.

### Statistical analysis

Statistical analysis of data was performed using SPSS (Version 29.0.2.0). Graphs were created using Microsoft Excel (Version 16.93.1). Normality of data was assessed using the Shapiro–Wilk test. Groups were compared using univariate analysis for variance (ANOVA) if normality was confirmed or the Kruskal–Wallis test if not. Frequencies were compared using a binary logistic regression including AAE. To correct for different AAE in the groups, Analysis of Covariance (ANCOVA) or Quade nonparametric ANCOVA was performed for the width of the ventricular system. Correlation between variables was tested using the Pearson correlation or partial correlation to correct for AAE. *P*-value was considered to be significant if <0.05. If post-hoc testing was performed, the *p*-value was corrected for multiple testing using the Bonferroni–Holm method. Due to the few cases of longitudinal data, no statistical tests were performed for analysis.

## Supplementary information

Supplementary information

## Data Availability

The datasets generated during the current study are not publicly available due to the presence of individual genetic and clinical information that could compromise participant privacy, but are available from the corresponding author on reasonable request.
