# Supplementary material for: Striatal hyperechogenicity as an ultrasound imaging marker for prodromal X-linked dystonia-parkinsonism
Source: NPJ Parkinsons Dis. 2026 Jun 3;12:133. doi: 10.1038/s41531-026-01418-4 (PMC13233847; doi:10.1038/s41531-026-01418-4)
Supplement: Supplementary file 1 — Supplementary information [file 41531_2026_1418_MOESM1_ESM.pdf]

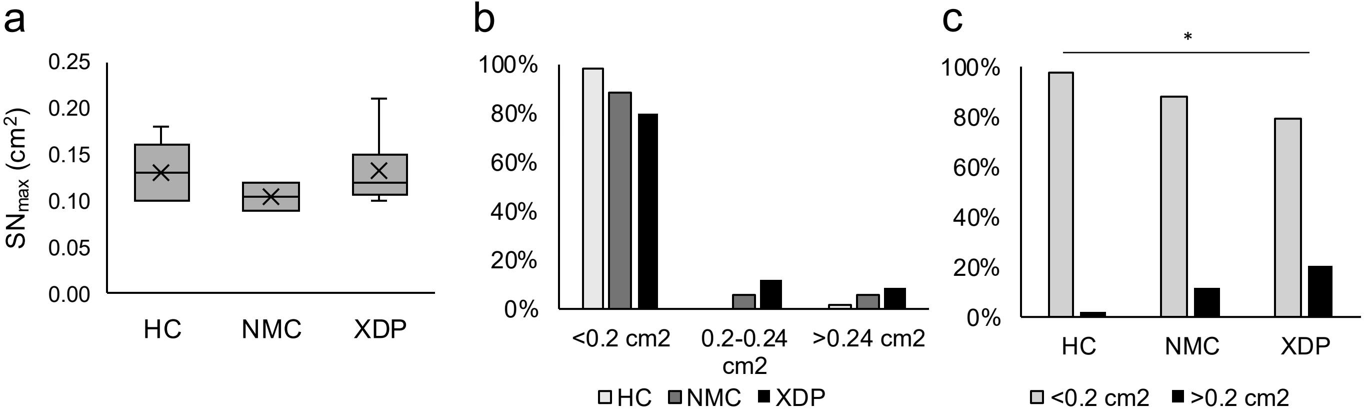

Supplement figure 1: a) Maximum size of in substantia nigra (SN_{max}) in cm^2 in regard to the group showing the 25th to 75th percentile as a box, the median as a line, the mean as an X, the minimum and maximum value in the range of 1.5x interquartile range as whiskers, and values outside of the 1.5x interquartile range as dots. b) Percentages of size of SNmax in regard to the group for <0.2 cm^2 (normal), 0.2 – 0.24 cm cm^2 (uncertain pathogenic), and >0.24 cm^2 (pathogenic). c) Percentages of size of SNmax in regard to the group. HC = healthy controls, NMC: non-manifesting carrier, XDP: patients with x-linked-Dystonia-Parkinsonism. *: p-value <0.05.
